# Coverage with Influenza, Respiratory Syncytial Virus, and Updated COVID-19 Vaccines Among Nursing Home Residents — National Healthcare Safety Network, United States, December 2023

**DOI:** 10.15585/mmwr.mm7251a3

**Published:** 2023-12-22

**Authors:** Hannah E. Reses, Heather Dubendris, Lori Haas, Kira Barbre, Sushmitha Ananth, Theresa Rowe, Elizabeth Mothershed, Elisha Hall, Ryan E. Wiegand, Megan C. Lindley, Sarah Meyer, Suchita A. Patel, Andrea Benin, Seth Kroop, Arjun Srinivasan, Jeneita M. Bell

**Affiliations:** ^1^Division of Healthcare Quality Promotion, National Center for Emerging and Zoonotic Infectious Diseases, CDC; ^2^Lantana Consulting Group, East Thetford, Vermont; ^3^Goldbelt C6, Chesapeake, Virginia; ^4^Leidos, Inc., Reston, Virginia; ^5^Immunization Services Division, National Center for Immunization and Respiratory Diseases, CDC.

**Summary**

**What is already known about this topic?**

Nursing home residents are vulnerable to infection with and complications from SARS-CoV-2, influenza, and respiratory syncytial virus (RSV). Vaccination reduces severe illness and death from these vaccine-preventable respiratory diseases.

**What is added by this report?**

As of December 10, 2023, 33% of nursing home residents were up to date with COVID-19 vaccination. Among residents at 20% and 19% of facilities that elected to report influenza and RSV vaccination coverage, respectively, 72% had received influenza vaccination, and 10% had received RSV vaccination.

**What are the implications for public health practice?**

There is an urgent need to protect nursing home residents against severe outcomes of respiratory illnesses by increasing vaccination against COVID-19 and influenza and discussing RSV vaccination with eligible residents.

## Abstract

Nursing home residents are at risk for becoming infected with and experiencing severe complications from respiratory viruses, including SARS-CoV-2, influenza, and respiratory syncytial virus (RSV). Fall 2023 is the first season during which vaccines are simultaneously available to protect older adults in the United States against all three of these respiratory viruses. Nursing homes are required to report COVID-19 vaccination coverage and can voluntarily report influenza and RSV vaccination coverage among residents to CDC’s National Healthcare Safety Network. The purpose of this study was to assess COVID-19, influenza, and RSV vaccination coverage among nursing home residents during the current 2023–24 respiratory virus season. As of December 10, 2023, 33.1% of nursing home residents were up to date with vaccination against COVID-19. Among residents at 20.2% and 19.4% of facilities that elected to report, coverage with influenza and RSV vaccines was 72.0% and 9.8%, respectively. Vaccination varied by U.S. Department of Health and Human Services region, social vulnerability index level, and facility size. There is an urgent need to protect nursing home residents against severe outcomes of respiratory illnesses by continuing efforts to increase vaccination against COVID-19 and influenza and discussing vaccination against RSV with eligible residents during the ongoing 2023–24 respiratory virus season.

## Introduction

Nursing home residents are at risk for becoming infected with and experiencing severe complications from respiratory viruses, including SARS-CoV-2 (*1*), influenza (*2*), and respiratory syncytial virus (RSV) (*3*). In 2023, the Food and Drug Administration approved the first two RSV vaccines for adults aged ≥60 years (*4*), making the 2023–2024 respiratory virus season the first in which vaccines against SARS-CoV-2, influenza, and RSV are simultaneously available in the United States. CDC recommends that all persons aged ≥6 months receive an updated (2023–24) COVID-19 vaccine dose* and a 2023–24 seasonal influenza vaccine.^†^ Among adults aged ≥60 years, CDC recommends RSV vaccine on the basis of shared clinical decision-making; residence in a nursing home is an important risk factor for RSV to consider in such decision-making (*4*). Since 2021, the Centers for Medicare & Medicaid Services (CMS) has required nursing homes to report weekly aggregate COVID-19 vaccination coverage among residents to CDC’s National Healthcare Safety Network (NHSN).^§^ Since October 21, 2023, nursing homes can also voluntarily report weekly, aggregate resident influenza and RSV vaccination coverage data to NHSN.^¶^ The purpose of this study was to assess COVID-19, influenza, and RSV vaccination coverage among nursing home residents during the ongoing 2023–24 respiratory virus season.

## Methods

### Data Collection

Nursing homes report COVID-19, influenza, and RSV vaccination coverage among residents who occupied a bed at the facility ≥1 day during the week of data collection. For each vaccine, nursing homes also report the number of residents who 1) received the most recently recommended vaccine, 2) had a medical contraindication to the vaccine, 3) declined the vaccine, or 4) had other or unknown vaccination status. NHSN defined up-to-date COVID-19 vaccination as the receipt of a 2023–2024 updated COVID-19 vaccine.**

### Data Analysis

Data reported from CMS-certified nursing homes for the week of December 10, 2023, (or the preceding week if data for December 10, 2023, were not available) were used for analysis.^††^ Because reporting of influenza and RSV vaccination coverage is voluntary, representativeness of facilities reporting these data was assessed by comparing important facility and county characteristics among reporting facilities and all facilities. Coverage estimates (percentage of residents vaccinated) for COVID-19, influenza, and RSV vaccine and 95% CIs were calculated using Poisson regression models. Residents reported to have a medical contraindication to a vaccine were subtracted from the corresponding denominator. For each vaccine, coverage was stratified by U.S. Department of Health and Human Services (HHS) region,^§§^ county-level social vulnerability index (SVI) tertile,^¶¶^ and facility size tertile.*** All analyses were conducted using SAS (version 9.4; SAS Institute). Nonoverlapping 95% CIs were considered to represent statistically significant differences (α = 0.05). This activity was reviewed by CDC, deemed not research, and conducted consistent with applicable federal law and CDC policy.^†††^

## Results

### Representativeness of Voluntary Influenza and RSV Reporters

Coverage with influenza and RSV as of December 10, 2023, was voluntarily reported by 3,046 (20.2%) and 2,939 (19.4%) of 15,113 CMS-certified nursing homes, respectively. Among these, the distributions of facilities by HHS region, SVI tertile, and facility size were comparable to distributions among all CMS-certified nursing homes enrolled in NHSN (Table).

**TABLE:** Estimates* of coverage with influenza, respiratory syncytial virus, and updated COVID-19 vaccines among nursing home residents — National Healthcare Safety Network, United States, December 2023^†^

Characteristic	Total no. of facilities^ §^ (column %)	Updated (2023–2024) COVID-19 vaccine			Influenza vaccine			RSV vaccine		
		Facilities reporting, no. (row %)	Total no. of residents	Coverage, % (95% CI)	Facilities reporting, no. (row %)	Total no. of residents	Coverage, % (95% CI)	Facilities reporting, no. (row %)	Total no. of residents	Coverage, % (95% CI)
**Total**	**15,113 (100.0)**	**14,646 (96.9)**	**1,240,163**	**33.1 (33.0–33.2)**	**3,046 (20.2)**	**247,280**	**72.0 (71.6–72.3)**	**2,939 (19.4)**	**238,449**	**9.8 (9.6–9.9)**
**HHS region^¶^**										
1	**826 (5.5)**	807 (5.5)	**75,349**	38.3 (37.8–38.7)	166 (5.4)	**13,421**	77.4 (75.9–78.9)	160 (5.4)	**13,055**	8.2 (7.7–8.7)
2	**969 (6.4)**	943 (6.4)	**136,743**	42.4 (42.0–42.7)	256 (8.4)	**33,738**	74.8 (73.8–75.7)	244 (8.3)	**31,891**	7.1 (6.9–7.4)
3	**1,383 (9.2)**	1,346 (9.2)	**135,526**	36.1 (35.8–36.4)	260 (8.5)	**24,557**	74.3 (73.2–75.4)	249 (8.5)	**23,593**	10.0 (9.6–10.4)
4	**2,682 (17.7)**	2,613 (17.8)	**241,100**	27.5 (27.3–27.7)	612 (20.1)	**55,566**	64.8 (64.2–65.5)	591 (20.1)	**53,802**	5.9 (5.7–6.1)
5	**3,285 (21.7)**	3,188 (21.8)	**240,295**	34.7 (34.5–35.0)	482 (15.8)	**34,473**	72.0 (71.1–72.9)	461 (15.7)	**33,141**	12.7 (12.3–13.1)
6	**2,050 (13.6)**	1,998 (13.6)	**148,859**	22.5 (22.3–22.8)	336 (11.0)	**23,244**	74.3 (73.2–75.4)	328 (11.2)	**22,483**	9.2 (8.8–9.6)
7	**1,430 (9.5)**	1,372 (9.4)	**81,667**	38.9 (38.4–39.3)	295 (9.7)	**16,292**	77.4 (76.1–78.8)	291 (9.9)	**15,994**	15.5 (14.9–16.1)
8	**592 (3.9)**	559 (3.8)	**34,722**	42.9 (42.2–43.6)	125 (4.1)	**7,235**	79.9 (77.9–82.0)	120 (4.1)	**7,039**	24.8 (23.7–26.0)
9	**1,469 (9.7)**	1,401 (9.6)	**119,888**	29.5 (29.2–29.8)	417 (13.7)	**33,181**	72.3 (71.4–73.2)	400 (13.6)	**31,932**	10.1 (9.8–10.5)
10	**427 (2.8)**	419 (2.9)	**26,014**	34.7 (34.0–35.4)	97 (3.2)	**5,573**	64.3 (62.2–66.4)	95 (3.2)	**5,519**	11.7 (10.9–12.7)
SVI**										
Low	**5,022 (33.4)**	4,882 (33.3)	**379,417**	38.5 (38.3–38.7)	963 (31.6)	**73,512**	73.7 (73.0–74.3)	930 (31.6)	**70,759**	10.7 (10.5–11.0)
Medium	**5,052 (33.6)**	4,890 (33.4)	**434,613**	32.3 (32.1–32.5)	968 (31.8)	**83,342**	71.6 (71.0–72.2)	932 (31.7)	**80,244**	10.0 (9.7–10.2)
High	**4,984 (33.1)**	4,823 (32.9)	**422,627**	29.1 (28.9–29.2)	1,098 (36.0)	**89,609**	70.9 (70.4–71.5)	1,060 (36.1)	**86,629**	8.7 (8.5–8.9)
**Facility size^††^**										
Small	**5,072 (33.6)**	4,810 (32.8)	**197,427**	37.3 (37.0–37.5)	1,156 (38.0)	**46,674**	77.4 (76.6–78.2)	1,116 (38.0)	**45,063**	15.3 (15.0–15.7)
Medium	**5,098 (33.7)**	4,996 (34.1)	**382,940**	32.3 (32.1–32.5)	985 (32.3)	**75,460**	72.2 (71.6–72.8)	953 (32.4)	**73,006**	9.3 (9.1–9.5)
Large	**4,943 (32.7)**	4,840 (33.0)	**659,796**	32.2 (32.1–32.4)	905 (29.7)	**125,146**	69.8 (69.3–70.2)	870 (29.6)	**120,380**	8.0 (7.8–8.1)

**Abbreviations:** HHS = U.S. Department of Health and Human Services; RSV = respiratory syncytial virus; SVI = social vulnerability index.

* Estimates of coverage (percentage of residents vaccinated) with influenza, RSV, and updated (2023–2024) COVID vaccines and 95% CIs were calculated using Poisson regression models. Residents who reported having a medical contraindication to each vaccine were subtracted from the corresponding denominator. Nonoverlapping 95% CIs were considered to represent statistically significant differences (α = 0.05).

^†^ Data reported from nursing homes certified by the Centers for Medicare & Medicaid Services for the week of December 10, 2023 (or the preceding week if data for December 10, 2023, were not available) were used for analysis.

^§^ Facilities were included if they were actively enrolled in the National Healthcare Safety Network Long-Term Care Component and had ever reported nonzero vaccination denominator data. Facilities were excluded from the coverage estimates if they reported zero residents or did not report data for either the week of December 10, 2023, or the week of December 3, 2023 (467 facilities were excluded for COVID-19; 12,067 were excluded for influenza; and 12,174 were excluded for RSV).

^¶^ https://www.hhs.gov/about/agencies/iea/regional-offices/index.html

** SVI categories are based on the tertile distribution of the SVI of the counties where the facilities are located (low: ≤0.38; medium: >0.38–<0.66; and high: ≥0.66). Fifty-five facilities are located in counties that have no assigned SVI score (COVID-19: 51 facilities; influenza: 17 facilities; and RSV: 17 facilities). https://www.atsdr.cdc.gov/placeandhealth/svi/index.html

^††^ Facility size category is based on the tertile distribution of the total number of residents per facility (small: ≤58 residents; medium: 59–94 residents; and large: ≥95 residents).

### Updated (2023–2024) COVID-19 Vaccination Coverage

COVID-19 vaccination coverage was reported by 14,646 (96.9%) nursing homes for the week of December 10, 2023. A total of 33.1% of nursing home residents were up to date with COVID-19 vaccination, ranging from 22.5% in HHS Region 6 to 42.4% and 42.9% in HHS regions 2 and 8, respectively. Up-to-date vaccination against COVID-19 varied by SVI tertile, with highest coverage (38.5%) in the least socially vulnerable counties and lowest coverage (29.1%) in the most vulnerable counties. Updated COVID-19 vaccination was higher in small facilities (37.3%) than in medium (32.3%) and large facilities (32.2%).

### Influenza Vaccination Coverage

Among the 20.2% of facilities that voluntarily reported influenza vaccination for the week of December 10, 2023, 72.0% of residents had received an influenza vaccine. Coverage ranged from 64.3% and 64.8% in HHS regions 10 and 4, respectively, to 79.9% in HHS Region 8. Influenza vaccination coverage was highest in the least socially vulnerable counties (73.7%) and lowest in the most socially vulnerable counties (70.9%). Influenza vaccination was higher in small facilities (77.4%) than in medium (72.2%) and large facilities (69.8%).

### RSV Vaccination Coverage

Among the 19.4% of nursing homes that voluntarily reported RSV vaccination data for the week of December 10, 2023, 9.8% of residents had received an RSV vaccine. Coverage ranged from 5.9% and 7.1% in HHS regions 4 and 2, respectively, to 15.5% and 24.8% in HHS regions 7 and 8, respectively. RSV vaccination coverage was highest in the least socially vulnerable counties (10.7%) and lowest in the most socially vulnerable counties (8.7%); coverage was highest in small facilities (15.3%) and lowest in large facilities (8.0%).

### Overall Vaccination Coverage by Region

For all three vaccines, coverage was highest overall in HHS Region 8. Specifically, North Dakota and South Dakota consistently reported high coverage with all three vaccines (Figure).

**FIGURE:**
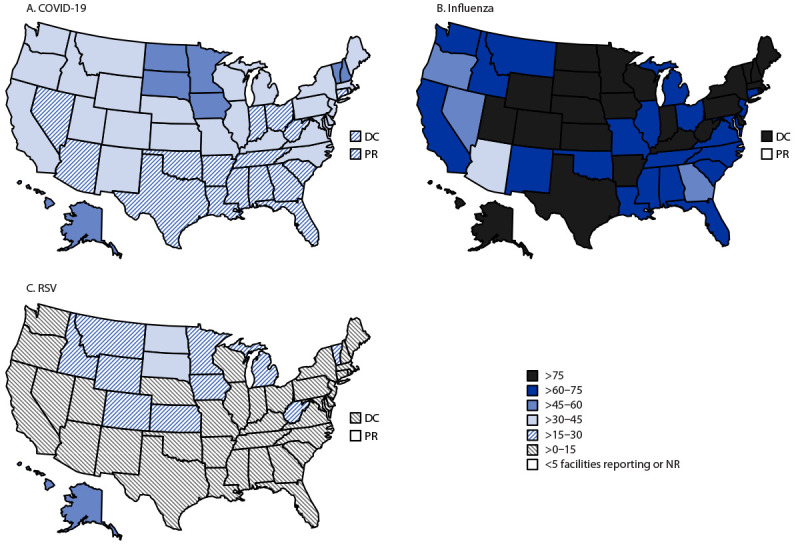
Estimates* of coverage with updated (2023–2024) COVID-19 vaccination (A), influenza vaccination (B), and RSV vaccination (C) among nursing home residents, by U.S. jurisdiction — National Healthcare Safety Network, United States, December 2023^†^ **Abbreviations: **DC = District of Columbia; NR = not reported; PR = Puerto Rico; RSV = respiratory syncytial virus**.** * Estimates of coverage (percentage of residents vaccinated) with influenza, RSV, and updated (2023-2024) COVID-19 vaccines were calculated using Poisson regression models. Residents who reported having a medical contraindication to each vaccine were subtracted from the corresponding denominator. ^†^ Data reported from nursing homes certified by the Centers for Medicare & Medicaid Services for the week of December 10, 2023, (or the preceding week if data for December 10, 2023, were not available) were used for analysis. Facilities were excluded from the coverage estimates if they reported zero residents or did not report data for either the week of December 10, 2023, or the week of December 3, 2023 (467 facilities were excluded for COVID-19; 12,067 were excluded for influenza; and 12,174 were excluded for RSV).

## Discussion

Although vaccination against SARS-CoV-2, influenza, and RSV reduces severe disease from these respiratory viruses in populations at high risk,^§§§^ coverage with each of the three vaccines, especially updated (2023–2024) COVID-19 and RSV vaccines, was low among nursing home residents. Compared with COVID-19 vaccination coverage among adults aged ≥65 years and RSV vaccination coverage among adults aged ≥60 years reported by the National Immunization Survey (NIS) Adult COVID-19 Module (37.4% and 17.0%, respectively), COVID-19 and RSV vaccination coverage reported to NHSN was lower among nursing home residents (33.1% and 9.8%, respectively). In contrast, influenza vaccination coverage among nursing home residents (72.0%) was slightly higher than that among the general adult population aged ≥65 years (69.3%) (*5*). Although data from NHSN and NIS cannot be directly compared because of different methodology and populations, these directional differences deserve further exploration.

Vaccine fatigue, defined as inaction toward vaccine information or instruction attributable to “perceived burden and burnout” (*6*), inaccurate health information, and vaccine hesitancy (*7*) contribute to lack of vaccine demand, especially in areas with a high SVI (*8*). For all three vaccines, coverage among nursing home residents was lowest in the most socially vulnerable counties. Lower coverage in areas with higher social vulnerability might be related to challenges to vaccine access and cost and payment barriers associated with COVID-19 vaccine commercialization.^¶¶¶^

The low RSV vaccination coverage relative to the other two vaccines might be driven by the relative recency of the recommendation, vaccine fatigue associated with the introduction of a fourth respiratory vaccine (in addition to influenza, COVID-19, and pneumococcal), implementation challenges with adding new vaccines, and the recommendation being based on shared clinical decision-making between a patient (or patient’s guardian) and a health care provider (*4*). Facilities have had limited time to train providers to implement a shared clinical decision-making recommendation and develop processes and policies to support RSV vaccine administration. Nursing home staff members might also be less familiar with the risk for RSV outbreaks and severe disease among residents (*9*). Increasing awareness of RSV as a cause of disease among nursing home residents might facilitate increased coverage.

In addition, these data highlight the success that can be achieved through surveillance and coordinated public health efforts to address barriers. During the 2023–2024 season, influenza vaccination coverage among nursing home residents was significantly higher than updated (2023–24) COVID-19 vaccination and RSV vaccination coverage. Annual influenza vaccination has been universally recommended since the 2010–11 influenza season,**** and CMS requires nursing homes to educate residents about and offer both influenza and COVID-19 vaccination. Notably, coverage with all three vaccines was highest in small nursing homes, suggesting that medical directors and other providers at these small facilities with lower patient-to-provider ratios might be best able to build trust with residents and families and mitigate barriers to vaccination coverage. HHS Region 8, driven largely by North Dakota and South Dakota, achieved relatively high coverage among nursing home residents with all three vaccinations because of robust relationships and frequent, persistent, clear communication among nursing homes, health care systems, state and local health departments, and pharmacies;^††††^ similar strategies might have the potential to improve vaccination coverage in other states. CDC is also engaged in efforts to increase vaccination coverage, including sharing NHSN surveillance data with state and local health departments and CMS Quality Innovation Networks-Quality Improvement Organizations to guide targeted outreach and educational efforts in nursing homes with lower vaccination coverage, contacting facilities with high coverage to learn about and promote successful strategies employed, working with national organizations that represent nursing homes to help educate staff members and residents, responding to barriers by developing a Healthcare Provider Toolkit to facilitate vaccination and conduct webinars with partners,^§§§§^ collaborating with CMS leaders to communicate reported billing barriers, and collaborating with CMS Quality Innovation Networks-Quality Improvement Organizations to increase vaccine confidence and demand.

### Limitations

The findings in this report are subject to at least three limitations. First, although it is mandatory for facilities to report COVID-19 vaccination coverage to NHSN, reporting of influenza and RSV vaccination coverage is optional, and the proportion of facilities reporting was low. Facilities that elected to report these data might be more likely to offer influenza or RSV vaccines. However, similarities in distribution of a small number of important facility demographics suggest that facilities voluntarily reporting these data might be representative of all facilities. Second, this analysis was conducted using aggregate, facility-level data reported to NHSN; therefore, vaccination coverage could not be directly examined by person-level covariates such as age, race, and ethnicity. Further, this limitation means that RSV vaccination coverage was calculated among all residents, not just the approximately 91% of residents aged ≥60 years (*10*). It is likely that RSV vaccination coverage among residents aged ≥60 years was higher than the overall coverage. Finally, NHSN does not collect data on the outcome of shared clinical decision-making discussions or reasons for declining vaccination.

### Implications for Public Health Practice

There is an urgent need to protect nursing home residents against severe outcomes of respiratory illnesses through continuing effective strategies to increase updated COVID-19 vaccination and influenza vaccination coverage and discussing RSV vaccination as an option among nursing home residents during the ongoing 2023–24 respiratory virus season. Health care providers should counsel residents that immunizations are the most effective way to prevent severe outcomes from COVID-19, influenza, and RSV and offer recommended immunizations.^¶¶¶¶^ It is important for nursing homes to collaborate with state and local health departments, federal agencies, and partners to address low vaccination coverage. Because vaccination coverage varied by vaccine type, region, SVI, and facility size, ongoing surveillance of vaccination coverage among nursing home residents remains essential to help guide timely efforts to increase vaccination in this population at high risk and address inequities.

## Footnotes

*
https://www.cdc.gov/coronavirus/2019-ncov/vaccines/stay-up-to-date.html

†
https://www.cdc.gov/flu/prevent/vaccinations.htm

§
https://www.federalregister.gov/documents/2021/05/13/2021-10122/medicare-and-medicaid-programs-covid-19-vaccine-requirements-for-long-term-care-ltc-facilities-and

¶
https://www.cdc.gov/nhsn/pdfs/covid19/ltcf/vax-rpv-protocol-ltc-residents-oct-2023-508.pdf

** NHSN defines up-to-date vaccination for surveillance purposes at the start of each quarter. https://www.cdc.gov/nhsn/pdfs/hps/covidvax/UpToDateGuidance-508.pdf

†† Facilities were included if they were actively enrolled in the NHSN Long-Term Care Component and had ever reported nonzero vaccination denominator data. Facilities were excluded from the coverage estimates if they reported zero residents or did not report data for either the week of December 10, 2023, or the week of December 3, 2023 (467 facilities were excluded for COVID-19; 12,067 were excluded for influenza; and 12,174 were excluded for RSV).

§§
https://www.hhs.gov/about/agencies/iea/regional-offices/index.html

¶¶ SVI considers socioeconomic status, household characteristics, race, ethnicity, housing type, and transportation. Counties in a lower SVI tertile are less socially vulnerable than are those in an upper SVI tertile. SVI categories were based on the tertile distribution of the SVI of the counties where the facilities are located (low: ≤0.38; medium: >0.38 to <0.66; and high: ≥0.66). Fifty-five facilities were excluded because they were located in counties that did not have an SVI score assigned. https://www.atsdr.cdc.gov/placeandhealth/svi/index.html

*** Facility size category was based on the tertile distribution of the total number of residents per facility (small: ≤58 residents; medium: 59–94 residents; and large: ≥95 residents).

††† 45 C.F.R. part 46.102(l)(2), 21 C.F.R. part 56; 42 U.S.C. Sect. 241(d); 5 U.S.C. Sect. 552a; 44 U.S.C. Sect. 3501 et seq.

§§§
https://www.cdc.gov/respiratory-viruses/tools-resources/health-care-providers.html

¶¶¶
https://www.healthaffairs.org/content/forefront/commercialization-covid-19-vaccines-raises-equity-concerns

****
https://www.cdc.gov/flu/professionals/vaccination/vax-summary.htm

†††† https://www.washingtonpost.com/opinions/2023/12/05/covid-vaccine-nursing-homes-dakota/; https://www.washingtonpost.com/opinions/2023/12/07/lessons-nursing-home-covid-vaccine/?utm_campaign=wp_follow_leana_s.+wen&utm_medium=email&utm_source=newsletter&wpisrc=nl-leanas.+wen

§§§§ CDC developed a toolkit for health care providers to prepare their patients for the fall and winter virus season (https://www.cdc.gov/respiratory-viruses/tools-resources/health-care-providers.html). This toolkit includes a guide for talking with patients about fall and winter viruses (https://www.cdc.gov/respiratory-viruses/tools-resources/downloads/HCP-conversation-guide-508.pdf), tips to prepare practices and patients for the fall and winter viruses season (https://www.cdc.gov/respiratory-viruses/tools-resources/downloads/6-tips-prepare-fall-winter-virus-season.pdf), an overview of immunization recommendations for the 2023–24 respiratory disease season (https://www.cdc.gov/respiratory-viruses/tools-resources/downloads/respiratory-disease-at-a-glance-508.pdf), and print materials for patients (https://www.cdc.gov/respiratory-viruses/tools-resources/downloads/protect-health-your-everything-infographic.pdf;  https://www.cdc.gov/respiratory-viruses/tools-resources/downloads/protect-health-your-everything-poster-11x17.pdf).

¶¶¶¶ https://emergency.cdc.gov/han/2023/han00503.asp
